# Analytical Validation of a New Enzymatic and Automatable Method for d-Xylose Measurement in Human Urine Samples

**DOI:** 10.1155/2017/8421418

**Published:** 2017-09-24

**Authors:** Israel Sánchez-Moreno, Carmen Monsalve-Hernando, Ana Godino, Luis Illa, María Jesús Gaspar, Guillermo Manuel Muñoz, Ana Díaz, José Luis Martín, Eduardo García-Junceda, Alfonso Fernández-Mayoralas, Carmen Hermida

**Affiliations:** ^1^Venter Pharma S.L., Azalea 1, Alcobendas, 28109 Madrid, Spain; ^2^Laboratorio de Análisis Clínicos, Hospital Universitario de la Princesa, Diego de León 62, 28006 Madrid, Spain; ^3^Werfen, Plaza de Europa 21-23, L'Hospitalet de Llobregat, 08908 Barcelona, Spain; ^4^Laboratorio de Análisis Clínicos, Hospital Universitario de Getafe, Carretera de Toledo Km 12,500, Getafe, 28905 Madrid, Spain; ^5^Siemens Healthcare Diagnostics S.L., Avenida Leonardo Da Vinci 15-17-19, Getafe, 28906 Madrid, Spain; ^6^Departamento de Química Bioorgánica, Instituto de Química Orgánica General (IQOG-CSIC), Juan de la Cierva 3, 28006 Madrid, Spain

## Abstract

Hypolactasia, or intestinal lactase deficiency, affects more than half of the world population. Currently, xylose quantification in urine after gaxilose oral administration for the noninvasive diagnosis of hypolactasia is performed with the hand-operated nonautomatable phloroglucinol reaction. This work demonstrates that a new enzymatic xylose quantification method, based on the activity of xylose dehydrogenase from* Caulobacter crescentus*, represents an excellent alternative to the manual phloroglucinol reaction. The new method is automatable and facilitates the use of the gaxilose test for hypolactasia diagnosis in the clinical practice. The analytical validation of the new technique was performed in three different autoanalyzers, using buffer or urine samples spiked with different xylose concentrations. For the comparison between the phloroglucinol and the enzymatic assays, 224 urine samples of patients to whom the gaxilose test had been prescribed were assayed by both methods. A mean bias of −16.08 mg of xylose was observed when comparing the results obtained by both techniques. After adjusting the cut-off of the enzymatic method to 19.18 mg of xylose, the Kappa coefficient was found to be 0.9531, indicating an excellent level of agreement between both analytical procedures. This new assay represents the first automatable enzymatic technique validated for xylose quantification in urine.

## 1. Introduction

Hypolactasia is defined as the deficiency in intestinal lactase (EC 3.2.1.23), which leads to an inefficient digestion of lactose [1]. This disorder is mainly genetically determined and affects more than half of the world population [2–4]. Hypolactasic individuals may develop nonspecific symptoms of lactose intolerance such as abdominal distension, audible intestinal sounds, bloating, and diarrhea after consumption of dairy products [1, 3, 5].

LacTEST® is a new noninvasive diagnostic method for hypolactasia based on the oral administration of gaxilose (4-*O*-*β*-d-galactopyranosyl-d-xylose), a synthetic disaccharide that is a close structural analogue of lactose and acts as substrate of intestinal lactase [6, 7]. Gaxilose is hydrolyzed into 2 physiological products: galactose and xylose. Galactose is transformed into glucose in the liver, while xylose, which is passively absorbed [8], is partially endogenously metabolized, with the rest appearing in blood and being finally excreted in urine. Total xylose amount determined in urine and xylose concentration in blood represent a measure of total lactase activity* in vivo* [6, 9–11].

Xylose can be detected in urine, plasma, and serum by different reported methods [12–18]. Among them, the most commonly used is the hand-operated phloroglucinol colorimetric method developed by Eberts et al. [13]. Since this method was not sensitive enough to detect the low amounts of xylose present in urine and blood after the oral ingestion of gaxilose, some modifications were introduced in the assay protocol. The resulting method was validated and was proved to be very sensitive and reliable [19]. Nevertheless, this methodology has represented a barrier for the use of the gaxilose test, since most of clinical laboratories are fully automated and reluctant to include any new manual analytical technique. The strong reaction conditions required in the phloroglucinol assay, which include the use of acid medium and incubation of the sample for 4 minutes at 100°C [13, 19], do not allow the automation of this reaction in standard clinical analyzers. The development of a new automatable xylose detection method was therefore required to expand the use of the gaxilose test.

To overcome these difficulties, we recently developed a new enzymatic quantification method for xylose measurement in urine human samples, based on the activity of the enzyme xylose dehydrogenase from* Caulobacter crescentus* (XylB) (EC 1.1.1.175) [20]. In a preliminary manual study, the linearity of the procedure was demonstrated to cover an adequate range of xylose concentrations, using both urine and buffer as sample matrix. Additionally, the technique was proved to be efficient and sensitive, representing a potentially good alternative to the phloroglucinol colorimetric reaction [20]. Taking these results into consideration, in this work we have optimized and validated the protocol in order to be implemented in three automated analyzers used in clinical laboratories. Furthermore, the level of agreement between both assays was evaluated comparing both analytical methods.

## 2. Materials and Methods

### 2.1. Principle of the New Enzymatic Quantification Method

**Figure 1:**
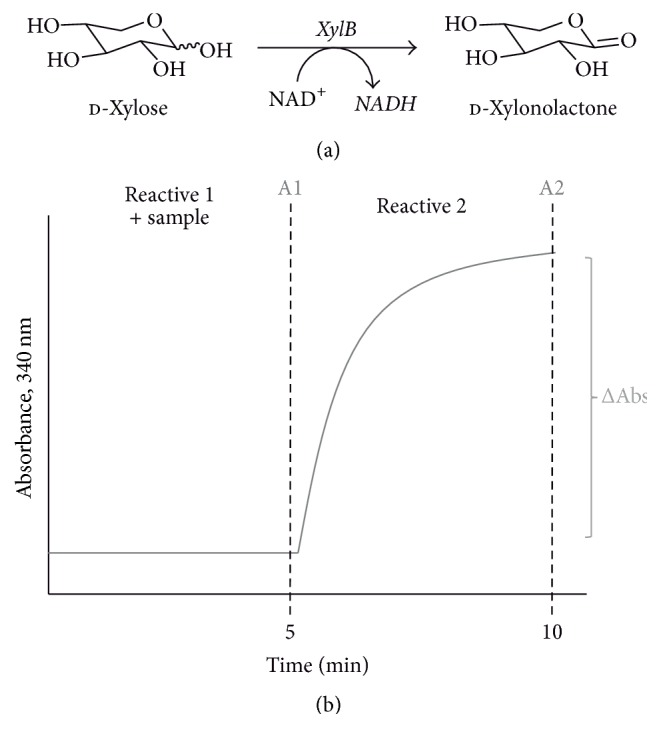
d-Xylose quantification with XylB. (a) Reaction catalyzed by XylB, in which produced NADH can be spectrophotometrically detected at 340 nm. (b) Typical assay profile obtained in the enzymatic d-xylose quantification method.

The enzyme XylB from* C. crescentus* catalyzes the specific oxidation of d-xylose to yield d-xylonolactone. As XylB is a NAD^+^-dependent dehydrogenase, this reaction requires a concomitant reduction of the cofactor to NADH (Figure 1(a)). Formation of NADH can be spectrophotometrically measured by the increase of the absorbance at 340 nm. Thus, absorbance differences will be directly proportional to the amount of d-xylose in the analyzed sample (Figure 1(b)).

### 2.2. Reagents

Sodium phosphate dibasic (NaH_2_PO_4_), NAD^+^, d-xylose, and phloroglucinol were purchased from Sigma-Aldrich. XylB was expressed and purified from* Escherichia coli* overproducer strain BL21(DE3)/pET-xylB-wt [20]. Two standard d-xylose solutions (FAR Diagnostics) were used as controls: control C1 (0.84 mg/dL) and control C2 (5.443 mg/dL).

### 2.3. Sample Collection and Preparation

Three kinds of samples were employed in this study: (i) solutions of d-xylose in aqueous buffer at different final concentrations, (ii) urine samples from healthy volunteers doped with different d-xylose concentrations, and (iii) urine samples from 224 patients to whom LacTEST 0.45 g had been prescribed.

Urine samples from healthy volunteers were collected as indicated in LacTEST 0.45 g summary of product characteristics (SPC) [21] but taking water instead of the diagnostic drug. Total excreted urine was collected during the following 5 hours. All the volunteers signed a written consent before urine collection. Urine samples from patients were collected during 5 hours after the administration of gaxilose, following also the specifications of LacTEST 0.45 g SPC [21].

Due to the lack of available commercial d-xylose quality controls mimicking the matrix of urine samples, the enzymatic method validation had to be performed with urine samples spiked with d-xylose. An initial pool was prepared with the urine samples of six healthy volunteers, ensuring that the matrix of the used samples was homogeneous. Various aliquots with different d-xylose concentrations were prepared from the initial pool using a standard certified commercial d-xylose from FAR Diagnostics. This standard solution (200 mg/dL) was diluted in urine or phosphate buffer (NaH_2_PO_4_ 50 mM, pH 8.0) to prepare the samples used in the study. All the collected and prepared samples were distributed, frozen, and stored at −80°C until use.

### 2.4. Assays Procedure

Enzymatic quantification of d-xylose in buffer or urine samples was carried out with the commercially available kit Xylossay® (Immunostep) following the manufacturer's instructions. The description of the two-reagent addition reaction for each automated analyzer is detailed in Supplemental Table 1 in Supplementary Material available online at https://doi.org/10.1155/2017/8421418. Briefly, Reactive 1 (R1) (2.46 mg/mL NAD^+^ in phosphate buffer) was mixed with the analyzed sample and incubated for 5 minutes. Then, Reactive 2 (R2) (0.024 mg/mL (60–80 U/L) XylB in phosphate buffer) was added and the mixture was incubated for 5 more minutes. Variation of absorbance at 340 nm (ΔAbs_340 nm_) before and after XylB addition was used to calculate the concentration of d-xylose in the sample. Calibration of the autoanalyzers was carried out using Xylossay® d-xylose standard solution (3.75 mg/dL) and water as sample blank (single-point calibration).

The colorimetric detection of d-xylose with phloroglucinol was carried out with Xylose Urine Kit (FAR Diagnostics) following the manufacturer's instructions and as previously reported [19]. Briefly, reactions were conducted in a final volume of 2.0 mL containing 1.9 mL of phloroglucinol color reagent, 50 *μ*L of sample, and 50 *μ*L of distilled water. Reactions were mixed, incubated for 4 minutes at 100°C, and cooled to room temperature in water. After mixing, absorbance was read at 554 nm. Calibration curves were obtained by serial dilution of the d-xylose standard solution (10 mg/dL) prepared by dissolving the d-xylose standard provided in the kit in distilled water.

### 2.5. Reagents Stability

The on-board stability of the enzymatic method reagents was evaluated along 39 days by measuring the d-xylose concentration in C1 and C2 controls (FAR Diagnostics) with the same reactives stored in Werfen ILab 600 autoanalyzer. This device was selected for the stability experiment because its reagent storage temperature (10°C) is the highest among the three evaluated autoanalyzers (the most restrictive storing condition).

### 2.6. Validation of the Enzymatic Method

Full validation of the method was carried out in Cobas c502 analyzer from Roche Diagnostics. The linear range was determined analyzing in triplicate 15 d-xylose concentrations (0–15 mg/dL) in buffer. Limit of detection (LoD) and limit of quantification (LoQ) were estimated from the regression parameters of the linearity experiment by the following statistical formulas: LoD = 3 × CV/*a*; LoQ = 10 × CV/*a*, where *a* is the slope and CV is the coefficient of variation [22, 23]. To establish the limit of blank (LoB) of the analytical method, two different blank solutions (distilled water and phosphate buffer) were analyzed 60 times each. LoB value was calculated using the following statistical formula: LoB = mean + 1.645 × SD [22]. Selectivity was assessed measuring in triplicate 6 individual urine samples from healthy volunteers who took water instead of the diagnostic drug. The carry-over was evaluated assaying two urine samples with d-xylose concentrations at 1.5 mg/dL (sample L, *N* = 11 replicates) and 9.0 mg/dL (sample H, *N* = 10 replicates), and it was calculated as previously reported [24].

Within-run precision and accuracy were, respectively, evaluated through the CV (%) and error (%) values calculated by assaying 20 times C1 and C2 controls, d-xylose samples in buffer at 1.5 mg/dL (S1), 3.75 mg/dL (S2), and 7.5 mg/dL (S3), and urine samples doped with d-xylose at 3.75 mg/dL (U5) and 7.5 mg/dL (U6), as well as measuring 10 times urine pools at 0.5 mg/dL (U1), 0.75 mg/dL (U2), 0.85 mg/dL (U3), and 0.95 mg/dL (U4). Between-run precision and accuracy assays were performed measuring C1, C2, U5, and U6 samples in duplicate along 20 consecutive days. Total imprecision was calculated as previously reported [25]. In addition, samples U1, U2, U3, and U4 were analyzed 60 times in different nonconsecutive days to calculate the lower limit of quantification (LLoQ), which is the lowest concentration of analyte in a sample which can be quantified reliably, with acceptable accuracy and precision [26].

Partial validations of the method were performed in the autoanalyzers Werfen ILab 600 and Siemens Dimension Vista 1500. Linearity, LoD, LoQ, and selectivity were calculated as previously explained. LoB was evaluated measuring distilled water (*N* = 12 replicates) and calculated as described above.

Precision and accuracy were studied as follows in Werfen ILab 600 device: C1 and C2 controls were, respectively, measured to calculate the within-run (*N* = 15 replicates) and between-run parameters (analyzed in duplicate along 10 consecutive days). Carry-over was evaluated in this analyzer by measuring three times a sequence of a sample with high concentration (37.5 mg/dL) followed by a sample with low concentration (0.075 mg/dL).

In Siemens Dimension Vista 1500 analyzer, within-run precision and accuracy data were obtained from the cross-validation experiment described below. Between-run precision and accuracy and the carry-over were not studied in this platform.

### 2.7. Cross-Validation Experiment

Four urine samples with different d-xylose concentrations (LL (0.55 mg/dL), LQ (1.5 mg/dL), MQ (6 mg/dL), and HQ (11.5 mg/dL)) were assayed in the three automated analyzers. Within-run precision and accuracy were calculated from these data, as well as the LLoQ for ILab 600 and Dimension Vista 1500 analyzers.

### 2.8. Comparison of Methods

The study of the correlation and the concordance of the two analytical methods was performed with 224 urine samples from patients to whom LacTEST 0.45 g had been prescribed from 10 different hospitals. Hypolactasia diagnosis for each patient had already been performed in each of the hospitals, quantifying xylose total amount in the collected urine by the phloroglucinol method. Patients with a xylose amount lower than 37.87 mg were diagnosed with hypolactasia [10, 11, 21]. Each hospital anonymously provided aliquots of urine samples, with the consent of the laboratory authorizing exclusively d-xylose measurement for the comparison of both analytical procedures. These aliquots had previously been stored at −20°C or −70°C in each center and were transported frozen in dry ice to the clinical laboratory of the Hospital de La Princesa in Madrid, where they were analyzed using both the phloroglucinol method and the previously validated enzymatic method with Cobas c502 analyzer from Roche. The results obtained by both techniques did not influence the diagnosis of the patients performed in each center.

### 2.9. Statistical Analysis

Linear regressions and validation statistical parameters were calculated with IBM SPSS Statistics computer program (IBM Corporation). Deming and Bland-Altman plots were generated with SigmaPlot 12.0 (Systat Software). Pearson's correlation coefficient and receiver operating characteristic (ROC) curves were obtained with XLSTAT software (Addinsoft).

## 3. Results and Discussion

### 3.1. Validation of the Enzymatic Method

The purpose of this study was to perform the analytical validation of the recently developed new enzymatic xylose quantification method in order to be implemented in automated equipment used in clinical laboratories. A complete validation of the new enzymatic assay was performed in Roche Cobas c502 analyzer. Additionally, partial validations were conducted in Werfen ILab 600 and Siemens Dimension Vista 1500 equipment. The parameters obtained are summarized in Table 1. Firstly, the linearity of the technique was demonstrated for xylose concentrations from 0.25 to 15 mg/dL (*R*^2^ = 0.9996–0.9999) (Supplemental Figure 1). The obtained values of LoD, LoQ, and LoB in each automated analyzer are summarized in Table 1, showing the high analytical sensitivity of the assay. Moreover, the carry-over was proved to reach a maximum value of 4.0%, although it was not determined in Siemens Dimension Vista 1500 device.

The European Medicines Agency (EMA) and Center for Drug Evaluation and Research (CDER) guidelines on bioanalytical method validation establish that the imprecision and inaccuracy of an analytical technique are clinically acceptable only if they are lower than 15% for samples with concentrations above the LLoQ [26, 27]. Within-run, between-run, and total CVs were lower than 3.7%, 11.5%, and 11.8%, respectively, in Roche Cobas c502 and 7.1%, 3.0%, and 7.7% in Werfen ILab 600. Regarding the accuracy of the method, within-run and between-run errors did not exceed 12.0% and 5.9%, respectively. For the Dimension Vista 1500 analyzer, only within-run imprecision and systematic error were measured, being lower than 13.6% and 9.7%, respectively (Table 1).

According to the EMA and CDER guidelines on bioanalytical method validation, the imprecision and inaccuracy should not exceed 20% for the concentration established as the LLoQ [26, 27]. The lowest xylose concentration determined with acceptable imprecision and inaccuracy values in Roche Cobas c502 (within-run and between-run CVs of 1.08% and 1.53%, resp., and within-run and between-run errors of 20.0%) was 0.85 mg/dL. Therefore, this concentration was established as the LLoQ of the technique in this analyzer.

In order to verify that all the samples have a similar behavior when analyzed in any of the three automated devices, four urine samples with different xylose concentrations were assayed in a cross-validation experiment (Table 2). LQ (1.5 mg/dL), MQ (6 mg/dL), and HQ (11.5 mg/dL) samples presented correct imprecision and inaccuracy values (<15%) [26, 27]. However, the behavior of LL (0.55 mg/dL) urine sample is different among the analyzers. In Siemens Dimension Vista 1500, which is the device with lower LoD and LoQ, the within-run error is 5.1%. In Roche Cobas c502, this value is 32.7%. This result was expected, since the LLoQ in this analyzer had previously been determined as 0.85 mg/dL. In Werfen ILab 600, the within-run error is 17.2%. Since this value is higher than 15% but lower than 20%, this accuracy is only acceptable if 0.55 mg/dL is considered as the LLoQ in this device [26, 27]. As no lower concentrations were measured, 0.55 mg/dL was also considered as the LLoQ in Siemens Dimension Vista 1500 analyzer (Table 1).

Finally, the EMA guideline establishes that the absence of interferences is accepted when the response is lower than 20% of the LLoQ [26]. The selectivity of the enzymatic technique was assessed measuring in triplicate 6 basal urine samples from healthy volunteers. The mean signals obtained were 0.16 ± 0.09 mg/dL, 0.10 ± 0.09 mg/dL, and 0.07 ± 0.05 mg/dL in Roche Cobas c502, Werfen ILab 600, and Siemens Dimension Vista 1500 analyzers, respectively, being lower than 20% of the LLoQ values and demonstrating the absence of interfering components in basal urine samples [26, 27]. Furthermore, selectivity of the enzymatic method in the presence of different monosaccharides was previously tested [20], showing that the assay displays high specificity for xylose over the other assayed sugars.

### 3.2. Stability of the Enzymatic Method Reagents

Stability of the freeze-dried and rehydrated XylB enzyme has been previously reported [20]. In this work, we evaluated the on-board stability of the reagents of the enzymatic xylose quantification kit. For this purpose, xylose concentrations in C1 and C2 controls were measured along 39 days with the same reactives stored in Werfen ILab 600 autoanalyzer. The obtained xylose concentrations for C1 and C2 controls were 0.77 mg/dL and 4.95 mg/dL on the first day and 0.66 mg/dL and 4.77 mg/dL on the last day, respectively, corresponding to recovery values of 93.1% and 100.3%.

### 3.3. Comparison of the Enzymatic and Phloroglucinol Methods

**Figure 2:**
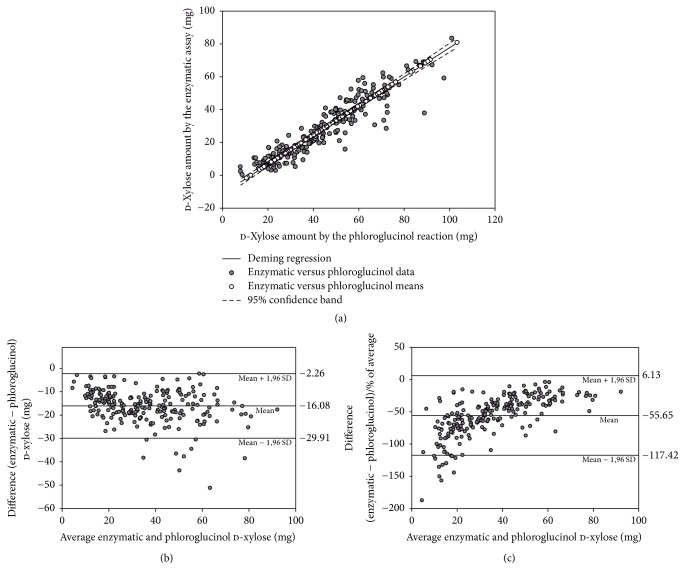
Comparison of the enzymatic assay versus the phloroglucinol method. The correlation and agreement between both methods was studied using (a) the weighted Deming regression, (b) the Bland-Altman plot, and (c) Bland-Altman plot of the differences between the enzymatic method and the phloroglucinol method expressed as percentages of the values on *x*-axis versus the mean of the two measurements.

To compare both analytical procedures, 224 urine samples of patients to whom LacTEST 0.45 g had been prescribed were assayed by both methods. The obtained total xylose amounts (expressed in mg) are detailed in Supplemental Table 2.  Figure 2(a) represents the weighted Deming regression of the results of the enzymatic assay versus the measurements of the phloroglucinol reaction. The slope is 0.8891 (95% CI: 0.8426–0.9356), the intercept is −11.2258 (95% CI: −13.4296–−9.0220), and Pearson's coefficient is 0.9321 (*P* < 0.0001), which indicates the existence of a very strong significant correlation between the two analytical techniques [28, 29]. The Bland-Altman plot is depicted in Figure 2(b). This analysis proves that the level of agreement between the two assays is low, since the mean bias obtained in the enzymatic method results is −16.08 mg. It also shows that the data present a wide dispersion, with mean ± 1.96 SD limits of −29.91 mg and −2.26 mg. To evaluate whether the bias between both analytical methods is constant or proportional to the average of the amount of xylose in the analyzed sample, the Bland-Altman plot was also represented with the difference expressed as percentage of the average (Figure 2(c)). The absolute value of the percentages decreases as the average of the amount of xylose in the sample augments, indicating that the difference between both techniques is constant [30]. However, the wide data dispersion (mean ± 1.96 SD limits of 6.13% and −117.42%) indicates a strong variation in the bias depending on the analyzed sample.

This bias may be due to a variable unspecific background detected by the phloroglucinol reaction, which is not present in all urine samples and may appear as a consequence of the strong reaction conditions required (acid medium and high temperature) [13, 19]. Moreover, the unspecific background in xylose detection is considerably reduced when the enzymatic method is employed, due to the lower matrix effect that urine samples produce in the photometric detection [20]. In fact, this variable background was observed during the first LacTEST® phase I clinical trial, in which detected xylose amounts in urine after the administration of placebo (water) to 12 healthy volunteers ranged from 1.93 to 22.64 mg, with a mean value of 12.92 ± 5.26 mg [10], suggesting the presence of an interfering component in some urine samples. This interfering substance remains unidentified. Despite this fact, the diagnostic performance of the gaxilose test with phloroglucinol xylose quantification was calculated in a phase IIb/III clinical trial, obtaining excellent sensitivity and specificity values [11].

**Figure 3:**
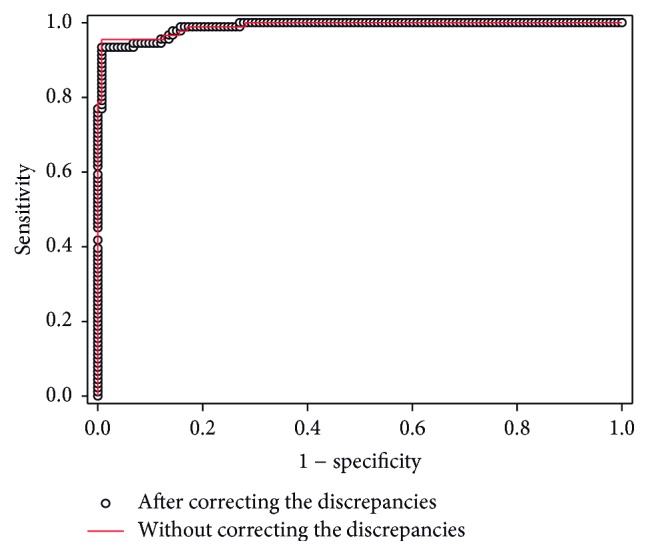
Concordance of the enzymatic assay versus the phloroglucinol method. The concordance between both methods was studied using the ROC curves of the enzymatic assay generated taking as reference the results of the phloroglucinol reaction. ROC curves were represented before and after correcting the discrepancies of patients 6 and 8.

Currently, the cut-off value of the total amount of xylose in urine determined by the phloroglucinol method after the administration of LacTEST 0.45 g is 37.87 mg. Patients with a xylose amount lower than this cut-off value are diagnosed with hypolactasia [10, 11]. The bias observed between the two analytical procedures represents 42.5% of this reference value, indicating that a new cut-off would be necessary to correctly diagnose hypolactasia when xylose is quantified with the new enzymatic method. Due to the wide dispersion observed in the differences between both techniques, the direct correlation between the two methods could not be straightly used to calculate the cut-off value of the enzymatic system. Consequently, the xylose cut-off of the enzymatic method was determined with the ROC curve using the phloroglucinol method as reference (Figure 3). The obtained area under the curve (AUC) was 0.989 (95% CI: 0.986–0.992) with a cut-off value of 19.18 mg. Hence, patients with a xylose amount determined by the enzymatic assay lower than 19.18 mg would be diagnosed with hypolactasia.

Among the 224 analyzed urine samples, we found 7 discrepancies between the results of both analytical techniques, corresponding to patients 6, 8, 62, 125, 145, 173, and 207 (Supplemental Table 2). In order to investigate whether these discrepancies could be resolved, we contacted the different physicians who had prescribed LacTEST 0.45 g to these patients to obtain information about their follow-up. Only the discrepancies corresponding to patients 6 and 8 could be corrected. The values obtained by the enzymatic and the phloroglucinol methods were 23.28 mg and 35.74 mg, respectively, for patient 6 and 20.65 mg and 24.14 mg, respectively, for patient 8 (Supplemental Table 2). According to the information provided by their physician, these patients were classified as normolactasic by the phloroglucinol determination conducted in their hospital, with xylose values of 39.5 mg for patient 6 and 52.3 mg for patient 8. The physician considered both patients as normolactasic, in agreement with the results obtained later with the new enzymatic procedure. In fact, these patients feel currently well, without symptoms of lactose intolerance. Therefore, taking the medical diagnosis into consideration, we changed the classification of patients 6 and 8 to normolactasic, being no longer discrepant to calculate the level of agreement between both quantification methods.

Among the 5 remaining discrepancies, those of patients 125 and 145 can also be explained. Xylose amounts determined by the enzymatic and phloroglucinol methods were 23.40 mg and 36.08 mg, respectively, for patient 125 and 22.85 mg and 35.68 mg, respectively, for patient 145 (Supplemental Table 2). These patients were consequently considered normolactasic with the enzymatic procedure and hypolactasic with the phloroglucinol assay. However, xylose values obtained with the phloroglucinol reaction are very close to the cut-off value of this method (37.87 mg) and within the range of measurement error of the phloroglucinol method (0.48–6.45%, as indicated in LacTEST 0.45 g SPC) [21]. On the other hand, the discrepancies found for patients 62, 173, and 207 (Supplemental Table 2) could not be attributed to any justifiable reason.

After correcting the two discrepancies of patients 6 and 8 (classification changed to normolactasic), we generated the corrected ROC curve (Figure 3). The cut-off value remained 19.18 mg, and the AUC was 0.991 (95% CI: 0.989–0.992). The concordance of both analytical techniques was assessed by calculating the Kappa coefficient, which yielded a value of 0.9531 (95% CI: 0.9125−0.9937). This value is considered indicative of excellent or almost perfect agreement between both analytical procedures [31, 32].

The new enzymatic method was developed to quantify xylose in urine samples after the administration of 0.45 g of gaxilose for hypolactasia diagnosis. Nevertheless, the excellent agreement between both techniques suggests that the enzymatic method could also replace the phloroglucinol reaction for xylose quantification in other clinical applications, such as the xylose malabsorption test. This test evaluates xylose absorption capacity of the small intestine through the oral administration and later quantification of this monosaccharide in urine [33]. However, the higher doses of xylose administered (5–25 g) and consequently the higher amount of monosaccharide excreted in urine would possibly require the dilution of urine samples or the adjustment of the enzymatic reaction.

## 4. Conclusions

In conclusion, the analytical validation of the new enzymatic xylose quantification method and the comparison with the phloroglucinol reaction demonstrated that the new procedure represents a good alternative to the phloroglucinol manual technique. Even though the analytical sensitivity of the latter was slightly better, with a LoQ of 0.46 mg/dL [19], the new enzymatic assay has the advantages of being automatable, not requiring the use of acids and heat, and avoiding possible errors due to sample manipulation. Furthermore, it represents the first enzymatic xylose quantification technique that has been validated and will facilitate the use of the gaxilose test in the clinical practice.

## Supplementary Material

The supplementary material file contains three items: Supplemental Figure 1 (linearity of the enzymatic D-xylose quantification method), Supplemental Table 1 (enzymatic xylose quantification general protocol used in Roche Cobas c502, Werfen ILab 600, and Siemens Dimension Vista 1500 automated analyzers), and Supplemental Table 2 (cumulated D-xylose values in patients' samples of urine collected during 5 hours after taking LacTEST 0.45).

## Figures and Tables

**Table 1:** Parameters obtained in the analytical validation of the enzymatic d-xylose quantification method.

Parameters	Cobas c502 (Roche)	ILab 600 (Werfen)	Dimension Vista 1500 (Siemens)
Range of linearity (mg/dL)	0.25 to 15	0.02 to 15	0.25 to 15
Linearity: *y* = *ax* + *y*_*o*_			
*R*^2^	0.9998	0.9996	0.9999
*a* (AU·dL/mg)	0.0610 ± 0.0004	0.088 ± 0.002	0.0285 ± 0.0002
*y*_*o*_ (mg/dL)	0.0006 ± 0.003	0.01 ± 0.01	0.003 ± 0.001
CV	0.0038	0.0144	0.0012
Limits			
LoB (mg/dL)	0.046	0.072	0.011
LoD (mg/dL)	0.186	0.490	0.130
LoQ (mg/dL)	0.623	1.640	0.420
LLoQ (mg/dL)	0.850	0.550	0.550
CV			
Within-run CV (%)	<3.7	<7.1	<13.6
Between-run CV (%)	<11.5	<3.0	ND
Total CV (%)	<11.8	<7.7	ND
Inaccuracy			
Within-run inaccuracy (%)	≤12.0	<9.0	<9.7
Between-run inaccuracy (%)	<5.9	<3.9	ND
Carry-over (%)	3.3	4.0	ND

LLoQ: lower limit of quantification; LoB: limit of blank; LoD: limit of detection; LoQ: limit of quantification; ND: not determined.

**Table 2:** Results of the cross-validation experiment in each automated analyzer.

	Cobas c502 (Roche)	ILab 600 (Werfen)	Dimension Vista 1500 (Siemens)
*Urine LL samples (0.55 mg/dL)*			
Mean (mg/dL)	0.730	0.644	0.522
SD	0.006	0.013	0.010
CV (%)	0.8	2.0	1.8
Inaccuracy (%)	32.7	17.2	5.1
*Urine LQ samples (1.50 mg/dL)*			
Mean (mg/dL)	1.680	1.590	1.356
SD	0.012	0.013	0.184
CV (%)	0.7	0.8	13.5
Inaccuracy (%)	12.0	6.0	9.6
*Urine MQ samples (6.00 mg/dL)*			
Mean (mg/dL)	6.270	6.776	6.032
SD	0.021	0.037	0.054
CV (%)	0.3	0.6	0.9
Inaccuracy (%)	4.5	12.9	0.5
*Urine HQ samples (11.50 mg/dL)*			
Mean (mg/dL)	11.880	11.748	12.022
SD	0.025	0.039	0.122
CV (%)	0.2	0.3	1.0
Inaccuracy (%)	3.3	2.2	4.5

## Nonstandard Abbreviations

XylB: Xylose dehydrogenase from* Caulobacter crescentus*
SPC: Summary of product characteristics
R1: Reactive 1
R2: Reactive 2
LoD: Limit of detection
LoQ: Limit of quantification
LoB: Limit of blank
LLoQ: Lower limit of quantification
ROC: Receiver operating characteristics
EMA: European Medicines Agency
CDER: Center for Drug Evaluation and Research
AUC: Area under the curve
ND: Not determined.
