# Supplementary material for: Analytical Validation of a New Enzymatic and Automatable Method for d-Xylose Measurement in Human Urine Samples
Source: Biomed Res Int. 2017 Sep 24;2017:8421418. doi: 10.1155/2017/8421418 (PMC5632886; doi:10.1155/2017/8421418)
Supplement: Supplementary file 1 — The supplementary material file contains three items: Supplemental Figure 1 (linearity of the enzymatic D-xylose quantification method), Supplemental Table 1 (enzymatic xylose quantification general protocol used in Roche Cobas c502, Werfen ILab 600, and Siemens Dimension Vista 1500 automated analyzers), and Supplemental Table 2 (cumulated D-xylose values in patients' samples of urine collected during 5 hours after taking LacTEST 0.45). [file 8421418.f1.pdf]

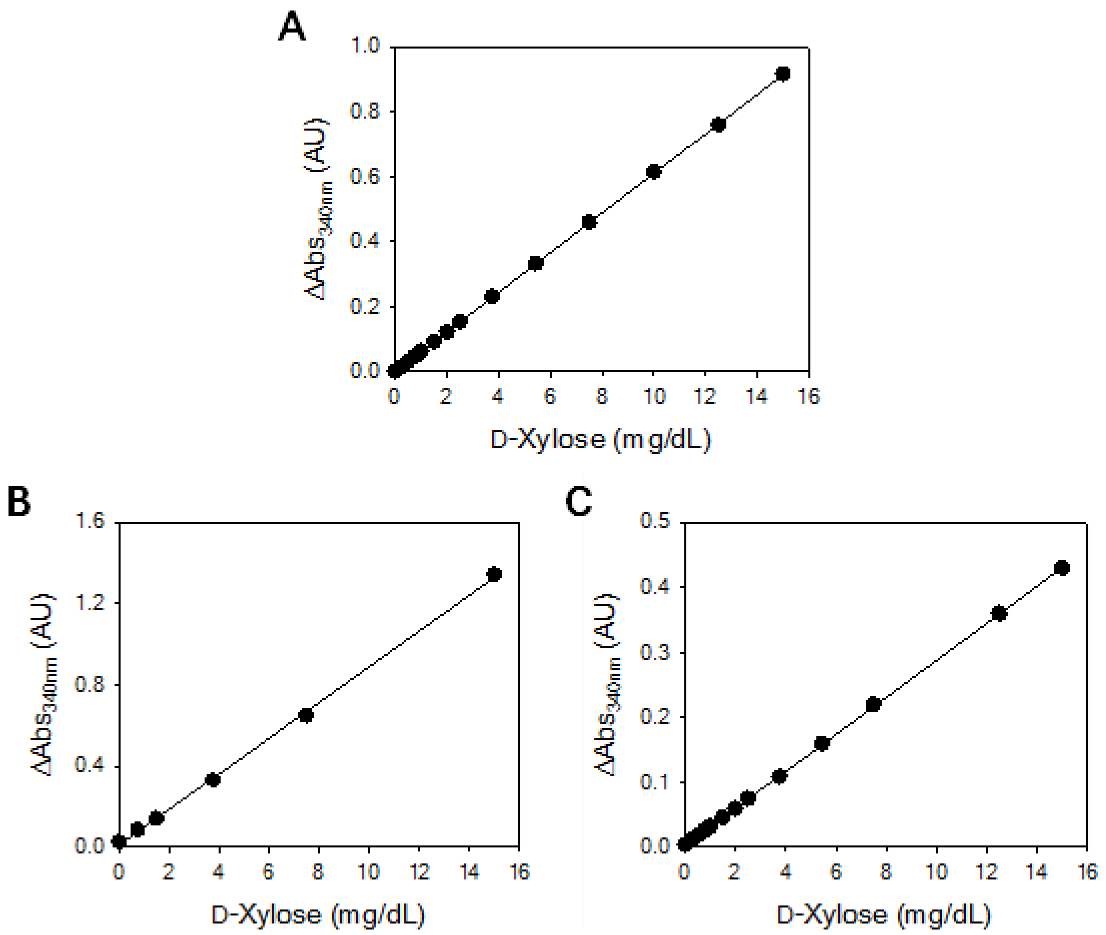

Supplemental Figure 1. Linearity of the enzymatic D-xylose quantification method. Samples with D-xylose concentrations ranging from 0 to 15 mg/dL were assayed in: (A) Roche Cobas c502 analyzer (15 concentrations by triplicate); (B) Werfen ILab 600 analyzer (6 concentrations by triplicate); and (C) Siemens Dimension Vista 1500 device (15 concentrations by duplicate).

Supplemental Table 1. Enzymatic xylose quantification general protocol used in Roche Cobas c502, Werfen ILab 600, and Siemens Dimension Vista 1500 automated analyzers.

Step	Reactive	Blank	Calibrator	Sample
1	Distilled water	F_1 x V_R (µL)	-	-
	REACTIVE 1 (phosphate buffer + NAD^+)	F_2 x V_R (µL)	F_2 x V_R (µL)	F_2 x V_R (µL)
	Sample (Urine)	-	-	F_1 x V_R (µL)
	Xylose Calibrator (3.75 mg/dL)	-	F_1 x V_R (µL)	-
Mix the reaction and incubate for 5 min. After this, read the absorbance (A1)				
2	REACTIVE 2 (Xylose dehydrogenase)	F_3 x V_R (µL)	F_3 x V_R (µL)	F_3 x V_R (µL)
Mix the reaction, incubate for 5 min and read the final absorbance (A2)				
Calculation method: two end-points.<br>Note: Each time xylose is measured in urine samples, xylose controls must be performed after calibration. These controls, one with low concentration and the second with high concentration, must be measured as if they were samples.				

V_R: final volume of the reaction in µL. It is the addition of the volumes of reactive 1, reactive 2 and distilled water (for the blank), sample or xylose calibrator.

V_R is 200 µL in Roche Cobas c502 and Werfen ILab 600 analyzers, and 145 µL in Siemens Dimension Vista 1500 device.

F_1, F_2 and F_3 are the dilution ratios of each reagent/sample with respect to the final volume of the reaction ($F_1+F_2+F_3 = 1$): $F_1 = 0.175$; $F_2 = 0.725$; $F_3 = 0.1$.

Supplemental Table 2. Cumulated D-xylose values (expressed in total mg) in patients' samples of urine collected during 5 hours after taking LacTEST 0.45 g. Samples were tested the same day both with the phloroglucinol method and the enzymatic method using Cobas c502 automated analyzer from Roche.

**n**	**Phloroglucinol Method (Total mg)**	**Cobas c502 Method (Total mg)**	**n**	**Phloroglucinol Method (Total mg)**	**Cobas c502 Method (Total mg)**
1	35.97	16.88	43	57.26	39.38
2	50.92	34.45	44	56.72	52.36
3	49.95	32.15	45	44.74	36.80
4	25.44	10.56	46	40.21	23.18
5	27.43	12.70	47	67.13	30.56
6	35.74	23.28	48	26.93	13.07
7	45.63	30.18	49	74.43	58.84
8	24.14	20.65	50	49.12	37.53
9	59.91	57.60	51	41.59	21.29
10	58.94	47.07	52	40.50	25.19
11	19.91	2.83	53	20.48	16.63
12	20.89	11.58	54	51.32	32.77
13	18.21	8.89	55	35.06	17.33
14	14.29	6.56	56	38.55	19.18
15	21.73	10.75	57	39.98	20.69
16	25.06	11.33	58	36.03	14.27
17	22.49	11.01	59	46.04	28.86
18	25.11	12.08	60	49.91	26.40
19	19.07	6.16	61	30.55	13.52
20	38.56	20.30	62	54.08	15.75
21	71.66	52.95	63	41.12	26.22
22	7.93	4.99	64	69.34	46.85
23	22.64	6.41	65	28.11	12.68
24	20.20	11.41	66	39.01	19.91
25	31.92	17.96	67	71.11	33.35
26	18.31	8.07	68	37.34	13.84
27	29.58	12.89	69	25.32	14.39
28	56.37	37.87	70	23.43	9.08
29	88.45	68.90	71	25.14	5.33
30	65.86	50.14	72	51.29	35.56
31	24.04	16.00	73	58.45	33.25
32	40.65	29.73	74	57.46	35.78
33	24.81	6.66	75	16.82	4.01
34	44.71	19.71	76	42.82	29.26
35	8.02	2.23	77	51.10	37.23
36	30.19	14.87	78	45.12	30.06
37	50.13	25.92	79	15.83	4.04
38	15.21	7.72	80	32.90	13.99
39	54.30	34.20	81	43.93	32.12
40	39.14	19.99	82	18.38	8.03
41	41.85	29.20	83	22.38	8.47
42	22.67	4.60	84	38.67	20.76

Supplemental Table 2. (continued)

n	Phloroglucinol Method Total mg	Cobas c502 Method Total mg	n	Phloroglucinol Method Total mg	Cobas c502 Method Total mg
85	35.14	16.88	133	16.51	5.50
86	27.62	7.11	134	25.48	11.22
87	58.15	39.28	135	72.55	42.00
88	52.00	39.55	136	18.64	6.94
89	38.57	19.96	137	44.07	26.87
90	56.72	45.39	138	56.04	33.63
91	51.56	20.94	139	60.36	42.12
92	60.14	52.05	140	45.58	35.75
93	50.16	31.52	141	71.26	47.04
94	51.91	47.03	142	43.66	30.23
95	52.78	35.63	143	31.14	7.56
96	40.22	24.68	144	61.94	59.30
97	54.51	44.82	145	35.68	22.85
98	63.01	50.90	146	86.70	66.90
99	33.64	17.54	147	31.79	10.56
100	72.72	48.78	148	42.61	19.55
101	81.17	66.45	149	71.54	54.70
102	72.52	49.70	150	33.32	18.15
103	44.57	25.32	151	26.31	18.30
104	44.33	23.29	152	70.71	62.21
105	50.28	39.66	153	55.75	45.23
106	38.18	19.20	154	64.61	47.18
107	85.28	69.09	155	31.82	12.22
108	50.83	27.20	156	35.54	9.28
109	61.70	46.23	157	50.03	40.66
110	47.21	25.19	158	34.46	18.53
111	44.18	22.52	159	28.38	7.18
112	54.67	45.79	160	54.02	38.78
113	47.78	38.29	161	73.54	59.71
114	29.33	15.27	162	18.92	10.20
115	45.44	40.80	163	40.74	24.57
116	13.99	10.44	164	26.90	14.31
117	22.43	6.29	165	17.72	7.16
118	28.10	15.98	166	20.34	16.75
119	49.71	33.71	167	28.29	17.36
120	97.60	59.02	168	63.03	36.98
121	43.37	28.85	169	69.23	46.53
122	21.59	6.73	170	21.46	8.01
123	44.08	22.79	171	29.46	13.82
124	33.75	15.09	172	39.12	26.64
125	36.08	23.40	173	34.85	28.43
126	22.55	13.13	174	14.69	10.44
127	62.05	42.04	175	70.94	51.19
128	72.19	28.41	176	54.99	45.64
129	53.31	32.40	177	16.61	7.45
130	52.39	34.98	178	21.47	3.03
131	32.12	5.18	179	62.74	58.13
132	52.86	34.55	180	77.76	56.64

Supplemental Table 2. (continued)

n	Phloroglucinol Method Total mg	Cobas c502 Method Total mg	n	Phloroglucinol Method Total mg	Cobas c502 Method Total mg
181	42.85	26.36	203	49.86	39.94
182	27.30	14.92	204	24.48	10.30
183	44.95	31.66	205	61.16	39.71
184	81.83	64.05	206	17.97	5.61
185	25.07	13.07	207	29.25	23.75
186	41.45	23.24	208	37.09	16.94
187	61.27	51.32	209	40.95	22.10
188	89.00	37.77	210	27.42	12.94
189	72.66	39.55	211	54.10	25.68
190	20.24	3.88	212	48.37	39.33
191	25.40	14.91	213	101.04	85.68
192	8.75	0.28	214	38.96	23.54
193	58.41	44.80	215	70.54	50.58
194	91.24	72.25	216	92.41	68.96
195	57.06	38.36	217	60.29	40.95
196	28.83	10.84	218	40.76	26.58
197	66.53	55.06	219	66.52	55.50
198	73.53	53.02	220	70.92	60.90
199	23.64	10.56	221	41.16	23.53
200	59.84	48.86	222	26.66	14.64
201	25.26	17.24	223	56.83	32.25
202	63.77	48.55	224	22.95	9.35
